# Patient-centred care in general dental practice - a systematic review of the literature

**DOI:** 10.1186/1472-6831-14-64

**Published:** 2014-06-05

**Authors:** Ian Mills, Julia Frost, Chris Cooper, David R Moles, Elizabeth Kay

**Affiliations:** 1 NIHR Academic Clinical Fellow in General Dental Practice and Honorary Lecturer, Plymouth University Peninsula Schools of Medicine & Dentistry, Plymouth, UK; 2 Institute of Health Research, University of Exeter Medical School, Exeter, UK; 3 Peninsula Technology Assessment Group, University of Exeter Medical School, Exeter, UK; 4 Oral Health Services Research and Education and Research, Plymouth University Peninsula Schools of Medicine & Dentistry, Plymouth, UK; 5 Peninsula Dental School and Dental Public Health, Plymouth University Peninsula Schools of Medicine & Dentistry, Plymouth, UK; 6 Plymouth University Peninsula Schools of Medicine & Dentistry, The John Bull Building, Research Way, Tamar Science Park, Plymouth PL6 8BU, UK

**Keywords:** Patient-centred care, Dentistry, General dental practice, Quality, Systematic review

## Abstract

**Background:**

Delivering improvements in quality is a key objective within most healthcare systems, and a view which has been widely embraced within the NHS in the United Kingdom. Within the NHS, quality is evaluated across three key dimensions: *clinical effectiveness, safety and patient experience*, with the latter modelled on the Picker Principles of Patient-Centred Care (PCC). Quality improvement is an important feature of the current dental contract reforms in England, with “patient experience” likely to have a central role in the evaluation of quality. An understanding and appreciation of the evidence underpinning PCC within dentistry is highly relevant if we are to use this as a measure of quality in general dental practice.

**Methods:**

A systematic review of the literature was undertaken to identify the features of PCC relevant to dentistry and ascertain the current research evidence base underpinning its use as a measure of quality within general dental practice.

**Results:**

Three papers were identified which met the inclusion criteria and demonstrated the use of primary research to provide an understanding of the key features of PCC within dentistry. None of the papers identified were based in general dental practice and none of the three studies sought the views of patients. Some distinct differences were noted between the key features of PCC reported within the dental literature and those developed within the NHS Patient Experience Framework.

**Conclusions:**

This systematic review reveals a lack of understanding of PCC within dentistry, and in particular general dental practice. There is currently a poor evidence base to support the use of the current patient reported outcome measures as indicators of patient-centredness. Further research is necessary to understand the important features of PCC in dentistry and patients’ views should be central to this research.

## Background

Patient-centred care (PCC) is recognised as a key dimension of quality within health care according to the Institute of Medicine [1]. The importance of patient-centred care has also been recognised by the Australian Commission on Safety and Quality in Healthcare [2], the King’s Fund [3], Agency for Healthcare Research and Quality [4] and the Picker Institute [5]. PCC is relevant throughout the world irrespective of the system of healthcare or the cultural differences [6]. The International Alliance of Patients’ Organizations (IAPO) represents patients of all nationalities and has a vision to ensure delivery of patient-centred care around the world.

Health services research suggests that PCC leads to enhanced patient satisfaction, improved outcomes, enhanced health status and reduced utilization of care [7-10]. It is also claimed that PCC can result in greater work satisfaction for professionals and reduced levels of medical litigation [11]. Such benefits are extremely desirable for patients, health professionals and commissioners and fully justify the current enthusiasm for the delivery of patient-centred care.

Recent healthcare reforms in the UK have focussed heavily on quality management, with assessment of quality incorporated into both primary and secondary care services. Quality has been defined in terms of *patient safety, clinical effectiveness *and the *experience of patients*[12]. These features have laid the foundations for indicators which will be used to measure improved quality of healthcare [13] with patient feedback playing an increasingly important role in measuring the level of quality delivered [14,15].

PCC is just as relevant within dentistry, although this may not be reflected in terms of the volume of current literature. The FDI World Dental Federation recognises the importance of quality assessment and improvement in dentistry, but reports that there is considerable variation across member countries in the approach to quality management [16]. The lack of an agreed definition of “quality in dentistry” is reported as a barrier to measuring quality and this is highlighted as key to future developments. The Dental Quality Alliance (DQA) is a group of professional organisations in the United States with a mission of advancing performance measures as a means to improving oral health, patient care and patient safety [17]. The DQA have embraced the definition and domains of quality as described by the Institute of Medicine and focus substantially on the importance of patient-centred care within dentistry [1].

In the UK, provision of quality care within the general dental services has been a long held aspiration, but designing an effective method of remuneration to ensure that this is delivered has proved a considerable challenge. In 2006 a new system was introduced which aimed to improve patient access, promote prevention and deliver quality. It is now generally agreed that the system failed to deliver on its key objectives [18,19]. These failings have been recognised [20,21] and there is now a greater emphasis on ‘quality’ in the UK. In February 2014 the Chief Dental Officer for NHS England launched “Improving Oral Health – A Call to Action” aimed at developing a long-term strategic plan for NHS dentistry. One of the key objectives within this plan is the delivery of high quality dental care [22]. A Dental Quality and Outcomes Framework (DQOF) to measure quality has already been developed in England and is currently being evaluated [23]. The current DQOF measure is based around 3 dimensions of quality: *Clinical Effectiveness*, *Patient Experience* and *Safety.*

### Patient-centred care

The term ‘patient-centred care’ is widely used but poorly understood. This can create confusion, where individuals may have vastly different values, expectations and perceptions of what is successful patient-centred care [24]. Various authors have attempted to define PCC in the medical, nursing and psychiatry literature [25-27] including such authorities as the Picker Institute, the Planetree Foundation, The King’s Fund and the Institute of Medicine [1,3,5,28]. PCC has also been studied extensively within primary care with benefits considered and definitions proposed [8,29,30].

There is widespread acceptance that a “patient-centred” approach to patient care is beneficial, although it is less clear what “patient-centred” actually means. Stewart et al [27]. recognised this and stated that;

*“Patient-centredness is becoming a widely-used but poorly understood concept in medical practice. It may be most commonly understood for what it is not – technology-centred, doctor-centred, hospital-centred, disease-centred”*[27]

Despite the apparent confusion around the term PCC, Stewart is confident that there is strong agreement internationally based on her own work and that of Little et al. [24,27]. Stewart et al. described six interactive components which formed the basis of the patient-centred clinical method, which supported a comprehensive and holistic approach to care [31]. This was further developed by the Picker Institute and subsequently the seminal publication by the Institute of Medicine in 2001 – “Crossing the Quality Chasm” [1].

In the UK, the NHS Patient Experience Framework [32] provides guidance on the elements of care considered to be critical to the patient experience based on primary research. The framework represents the key dimensions of PCC and is closely aligned to the Picker Principles of Patient-Centred Care [5]. The Department of Health state that the Patient Experience Framework “*provides a common evidence based list of what matters to patients*”.

There are many definitions of PCC, but the Institute of Medicine version seems to have gained widespread acceptance.

*“provision of care that is respectful of and responsive to individual patient preferences, needs, and values and ensuring that patient values guide all clinical decisions”*[1]

In view of the importance of PCC, we considered that a systematic literature review was indicated to assess the current literature within dentistry.

## Methods

### Aim of literature review

A systematic review of the literature was undertaken with the following key objective:

What core elements of patient-centred care have previously been described in relation to dentistry that are based on primary research?

### Search strategy

A methodical approach based on the Centre for Reviews and Dissemination guidance for undertaking reviews in health care [33] was adopted using electronic databases to search the literature, supplemented by hand searching and cross-referencing [34] (Additional file 1).

Databases searched included PubMed, MEDLINE, PsychINFO, SocINDEX, Dentistry & Oral Sciences Source, The Cochrane Library and CINAHL. Search terms were based on key words and phrases such as ‘patient-centred care’, ‘person centred care’, ‘person focussed care’, ‘oral’, ‘dental’ and ‘dentistry’. The PCC terms and dental terms were then combined as a separate search to identify articles associated with PCC in dentistry. MeSH terms were also used for both ‘patient-centred care’ and ‘dentistry’, and details of the search criteria are demonstrated in Additional file 2.

Additional databases were subsequently included within the search and these involved NHS Evidence, HMIC, Cochrane Oral Health and Web of Science. Further searching was conducted on the internet via search engines such as Google and Google Scholar, and specific websites were used to search for articles or policy documents including the Kings Fund, Picker Institute, Planetree Foundation and the Institute for Patient and Family-Centered Care.

It is recognised that protocol driven search strategies do not always manage to identify all the relevant sources of literature, irrespective of the number of databases included within the search [35]. The protocol driven search strategy was therefore supplemented with additional search techniques including one generation backward searching, forward citation chasing and personal communication [34]. These additional searches identified a number of additional papers, which had not been found in the original search of the main databases. This fully justified our comprehensive approach to the search strategy and highlights the importance of including additional search techniques to identify relevant literature.

### Inclusion/Exclusion

Inclusion/exclusion criteria were applied to the articles identified from the literature search and the details are included in Table 1. We were specifically interested in assessing the evidence base for PCC within general dental practice and consideration was given to limit the literature search to such studies. However, it was felt that this would be too specific and potentially exclude some relevant publications. A decision was therefore made to include all literature investigating the concept of “patient-centred care” within any aspect of dentistry.

**Table 1:** Details of inclusion/exclusion criteria

**Inclusion**	**Exclusion**
Published between January 1970 and February 2013	Published prior to 1970
Published in English and full-text accessible	Articles which were not available in English or full text unavailable
Content directly relevant to dentistry or related disciplines	Not concerned with the field of dentistry
Articles investigating the conceptual framework or dimensions of patient-centred care	Articles not directly related to “patient-centred care”
Any publication undertaking primary research into PCC irrespective of type of study design	Opinion articles, reviews or reports of non-original research

The focus of this review was on determining the evidence base to support an understanding of PCC within dentistry. It was therefore solely concerned with primary research describing the dimensions or features of PCC. All forms of publication or article were included irrespective of the study design, the setting or the demographics. Opinion articles, reviews and articles reporting non-original research were excluded. Additional inclusion criteria were applied to ensure that articles were available in English language and published between January 1970 and February 2013. It was considered unnecessary to obtain articles prior to 1970 as it is generally accepted that the concept of PCC was originally introduced by Enid Balint as “patient-centred medicine” in 1969 [36].

A two-stage process was used during screening. Stage 1 involved initial screening of the title and abstract against the inclusion criteria. At this stage if there was any degree of uncertainty over the applicability of the publication it was automatically included and the full text article assessed. Stage 2 involved screening of the full text articles against the predetermined inclusion criteria with eligible studies for review identified at this stage. The inclusion criteria were applied as an absolute, and all aspects of the criteria needed to be fulfilled for inclusion within the review. The key requirements for inclusion were:

• Publication of primary research study within peer-reviewed journal

• Research related to dentistry

• Research findings describing a conceptual framework for patient-centred care or its constructs.

### Search outcome

Our search yielded 203 citations. Of these 203 papers, the initial electronic search accounted for 162 articles, with additional searching techniques identifying the remaining 41 papers. At Stage 1 screening, the 203 papers were assessed by the lead author (IJM) against the pre-determined selection criteria based on the information available in the title and abstract, if available. One hundred and fifty five papers were excluded at this stage, as they were not relevant to the area of research interest. The full text articles of the remaining 48 papers were obtained and assessed (Stage 2). Assessment of each article against the screening criteria was undertaken by the first author (IJM) with the advice and support of the rest of the research team. These 48 papers originated from various countries although a significant proportion of the literature (54%) was published from North America, with a further 27% from the UK.

Of these 48 papers, only 3 publications were considered to have met the inclusion criteria in full and were directly relevant to the aims of this literature review. Of the potentially eligible 48 citations, 29 were based on personal opinion or review and encompassed a wide range of topics where the term “patient-centred” had been applied. These articles were excluded as they did not undertake primary research into PCC in relation to dentistry or review the findings of such research. The remaining 19 articles undertook primary research with 11 papers using quantitative methodology and the remaining 8 utilising qualitative methods. Sixteen of these articles (11 quantitative and 5 qualitative) did not meet the inclusion criteria in terms of their research aims and were subsequently excluded.

### Quality appraisal

There are various quality appraisal tools which can be used to assess the quality of the literature depending on the type of research undertaken. There is no universally agreed quality appraisal tool for qualitative research [37], but the Critical Appraisal Skills Programme (CASP) for qualitative research [38] is considered to be a suitable tool for quality appraisal in the oral health field [39]. The quality appraisal of the 3 eligible papers was undertaken by the lead author (IJM) in discussion with the other authors. The quality assessment was based on aspects of the research team, study methods, context of the study, findings, analysis and interpretations [40].

### Data extraction

Data extraction was primarily concerned with assessment of the evidence base of the literature and identification of the dimensions or features of PCC, which had been described. The features of PCC described within the texts were coded and examined for similarities and variations between each other. They were also compared to existing seminal texts [1,5] reporting dimensions of PCC within the broader literature.

## Results

Almost two-thirds of the literature identified following stage 1 screening was based on opinion or review, with the remainder of the articles published as primary research. Many of the opinion papers identified within this literature review describe features of PCC as reported by Gerteis [41] and published in the seminal text “Crossing the Quality Chasm” by the Institute of Medicine in 2001 [1]. The opinion papers identified within this literature review reveal a strong correlation with the dimensions of PCC as described by the Institute of Medicine, although it is important to appreciate that the views expressed have not been formulated on evidence based dental research.

Of the nineteen primary research papers, only three described PCC and its dimensions through original research. The other sixteen research papers were excluded as they did not explore the nature of PCC. Eleven of the excluded articles were concerned with investigation of “patient-centred” outcome measures rather than PCC and were therefore not relevant to this review. Four articles assessed the delivery of PCC, or a dimension thereof, by using measurement tools previously described in the medical literature. The remaining article used the term “patient-centred” purely as a descriptor and was not concerned with the investigation of any aspect of PCC (See Table 2).

**Table 2:** Details of 48 papers identified from literature review and reason for exclusion

	**Authors**	**Title**	**Journal**	**Date**	**Type of article**	**Reason for exclusion**
1	Bauer, J., et al.	Interdisciplinary resources optimize Evidence-Based Dental Practice	J Evid Based Dent Pract	2005	**O**	NOT PRIMARY RESEARCH
2	Bedos, C., et al.	Patient-centred approaches: new models for new challenges	J. of the Canadian Dental Assoc	2011	**O**	NOT PRIMARY RESEARCH
3	Brennan, M., et al.	Patient satisfaction and oral health-related quality of life outcomes of implant overdentures and fixed complete dentures	Int J Oral Maxillofac Implants	2010	**QUAN**	Concerned with patient-centred outcomes and patient satisfaction rather than PCC
4	Chapple, H., et al.	Exploring dental patients’ preferred roles in treatment decision-making - a novel approach	British Dental Journal	2003	**QR**	Focuses on SDM rather than PCC
5	Cobb, D. S.	Patient-centered care in an academic health center: faculty perspective	J Dent Educ	1996	**O**	NOT PRIMARY RESEARCH
6	Cohen, L. A., et al.	Comparison of patient centeredness of visits to emergency departments, physicians, and dentists for dental problems and injuries	J Am Coll Dent	2010	**QUAN**	Assessment of level of PCC following dental injury using NHQR questionnaire rather than defining dimensions of PCC
7	Dierens, M., et al.	Patient-centered outcome of immediately loaded implants in the rehabilitation of fully edentulous jaws	Clinical Oral Implants Research	2009	**QUAN**	Concerned with patient-centred outcomes rather than PCC
8	Eriksen, H. M., et al.	A patient-centred approach to teaching and learning in dental student clinical practice	Eur J Dent Educ	2008	**O**	NOT PRIMARY RESEARCH
9	Fernandes, L., et al.	Patient-centered evaluation of orthodontic care: a longitudinal cohort study of children’s and parents’ attitudes	Am J. of Ortho & Dentofacial Orthopedics	1999	**QUAN**	Concerned with patient-centred outcomes rather than PCC
10	Formicola, A. J., et al.	Evolution of dental school clinics as patient care delivery centers	J Dent Educ	2006	**R**	NOT PRIMARY RESEARCH
11	Formicola, A. J., et al.	Evolution of dental school clinics as patient care delivery centers	J Dent Educ	2008	**R**	NOT PRIMARY RESEARCH
12	Freeman, R.	Communicating with children and parents: recommendations for a child-parent-centred approach for paediatric dentistry	Eur Arch Paediatr Dent	2008	**R**	NOT PRIMARY RESEARCH
13	Gerbert, B., et al.	The provider-patient relationship in academic health centers: the movement toward patient-centered care	J Dent Educ	1996	**O**	NOT PRIMARY RESEARCH
14	Gold, S. A.	Managing the patient-centered practice	J Calif Dent Assoc	2000	**O**	NOT PRIMARY RESEARCH
15	Hegarty, A. M., et al.	Patient-centred outcome measures in oral medicine: are they valid and reliable?	Int J Oral Maxillofac Surg	2002	**QUAN**	Study to compare patient-centred outcome measures and not PCC
16	Heymann, H. O.	The “patient-centered” practice	J Esthet Restor Dent	2008	**O**	NOT PRIMARY RESEARCH
17	Hietasalo, P., et al.	Cost-effectiveness of an experimental caries-control regimen in a 3.4-yr randomized clinical trial among 11-12-yr-old Finnish schoolchildren	European Journal Of Oral Sciences	2009	**QUAN**	“Patient-centred” used solely as a descriptive term and no reference to PCC.
18	Jones, C., et al.	Darzi and Dentistry:The Emerging Quality Agenda for GDPs in England	Dental Update	2009	**O**	NOT PRIMARY RESEARCH
19	Hislop, J. M.	Chapter 7: PATIENT-CENTERED COMMUNICATION	Dental Communication Unlimited	1999	**O**	NOT PRIMARY RESEARCH
20	Kalkwarf, K. L.	Patient-centered care in an academic health center: an administrator’s perspective	J Dent Educ	1996	**O**	NOT PRIMARY RESEARCH
21	Kalkwarf, K. L.	Patient-centered care and today’s dental practice	J Am Coll Dent	1997	**O**	NOT PRIMARY RESEARCH
22	Kulich, K. R., et al.	A qualitative analysis of patient-centered dentistry in consultations with dental phobic patients	Journal Of Health Communication	2003	**QR**	INCLUDED
23	Levine, R. A.	A patient-centered periodontal program for the 1990s, Part II	Compendium	1990	**R**	NOT PRIMARY RESEARCH
24	Levine, R. A.	A patient-centered periodontal program for the 1990s, Part I	Compendium	1990	**R**	NOT PRIMARY RESEARCH
25	Loignon, C., et al.	Providing humanistic care: dentists’ experiences in deprived areas	Journal of Dental Research	2010	**QR**	INCLUDED
26	Logan, H. L.	What does patient-centered care mean in an academic health center? A conclusion	J Dent Educ	1996	**O**	NOT PRIMARY RESEARCH
27	Logan, H. L.	What does patient-centered care mean in an academic health center? An introduction	J Dent Educ	1996	**O**	NOT PRIMARY RESEARCH
28	Madhan, B., et al.	Attitudes of postgraduate orthodontic students in India towards patient-centered care	J Dent Educ	2011	**QUAN**	Assessment of level of PCC of ortho students using PPOS questionnaire rather than defining dimensions of PCC
29	Mahajan, A., et al.	A Patient-Centered Clinical Evaluation of Acellular Dermal Matrix Graft in the Treatment of Gingival Recession Defects	Journal of Periodontology	2007	**QUAN**	Concerned with patient-centred outcomes and patient satisfaction rather than PCC
30	McGrath, C., et al.	Patient-centred outcome measures in oral surgery: validity and sensitivity	Br J Oral Maxillofac Surg	2003	**QUAN**	Concerned with patient-centred outcomes rather than PCC
31	McGrath, C., et al.	Patient-centred outcome measures for oral mucosal disease are sensitive to treatment	Int J Oral Maxillofac Surg	2003	**QUAN**	Concerned with patient-centred outcomes rather than PCC
32	McGrath, C., et al.	Patient-centred measures in dental practice: 2. Quality of life	Dent Update	2007	**R**	NOT PRIMARY RESEARCH
33	McNair, A., et al.	A qualitative study to develop a tool to examine patients’ perceptions of NHS orthodontic treatment	Journal of Orthodontics	2006	**QR**	Concerned with patient-centred outcomes and patient satisfaction rather than PCC
34	Miles, L. L.	The patient-centered practice	J Calif Dent Assoc	2000	**O**	NOT PRIMARY RESEARCH
35	Newsome, P., et al.	Patient-centred measures in dental practice: 1. An overview	Dent Update	2006	**R**	NOT PRIMARY RESEARCH
36	Newsome, P., et al.	Patient-centred measures in dental practice: 3. Patient satisfaction	Dent Update	2007	**O**	NOT PRIMARY RESEARCH
37	Ni Riordain, R., et al.	A patient-centered approach to developing a quality-of-life questionnaire for chronic oral mucosal diseases	Oral Surg, Oral Med, Oral Path, Oral Rad & Endo	2011	**QR**	Concerned with patient-centred outcomes rather than PCC
38	Nimmo, A., et al.	Patient-centered competency-based education in fixed prosthodontics	J Prosthodont	1996	**R**	NOT PRIMARY RESEARCH
39	Pastagia, J., et al.	The effect of patient-centered plaque control and periodontal maintenance therapy on adverse outcomes of periodontitis	The Journal Of Evidence-Based Dental Practice	2006	**R**	NOT PRIMARY RESEARCH
40	Phillips, C.	Patient-centered outcomes in surgical and orthodontic treatment	Semin Orthod	1999	**O**	NOT PRIMARY RESEARCH
41	Roskell, C., et al.	Developing patient-centred care in health professionals: reflections on introducing service-learning into the curriculum	Int J of Therapy & Rehab	2012	**QR**	Pilot study to promote PCC rather than explore dimensions
42	Sachdeva, R. C.	SureSmile technology in a patient--centered orthodontic practice	Journal Of Clinical Orthodontics: JCO	2001	**O**	NOT PRIMARY RESEARCH
43	Scambler, S., et al.	Professional attitudes towards disability in special care dentistry	Journal of Disability & Oral Health	2011	**QR**	INCLUDED
44	Shenkin, J. D.	Patient-Centered Caries Prevention in Children may be More Cost-Effective in the Long Term than Traditional Dental Care	J Evid Based Dent Pract	2011	**R**	NOT PRIMARY RESEARCH
45	Stutts, L. A., et al.	Patient-Centered Outcome Criteria for Successful Treatment of Facial Pain and Fibromyalgia	Journal of Orofacial Pain	2009	**QUAN**	Concerned with patient-centred outcomes rather than PCC
46	Travess, H. C., et al.	The development of a patient-centered measure of the process and outcome of combined orthodontic and orthognathic treatment	J Orthod	2004	**QR**	Concerned with patient-centred outcomes rather than PCC
47	Tresolini, C. P.	Health care relationships: instruments for effective patient-focused care in the academic health center	J Dent Educ	1996	**O**	NOT PRIMARY RESEARCH
48	Weinstein, P.	Provider versus patient-centered approaches to health promotion with parents of young children: what works/does not work and why	Pediatr Dent	2006	**O**	NOT PRIMARY RESEARCH

Key – type of article

**QUAN** - Primary research using **quantitative** methodology.

**QR -** Primary research using **qualitative** methodology.

**O** - Opinion article.

**R** - Narrative review.

Only 3 papers [42-44] fulfilled the predetermined inclusion criteria and provided data and evidence describing the key features of patient-centred care within dentistry. The literature review demonstrates that there have not been any published studies on patient-centred care within general dental practice. The 3 relevant papers are included in Table 3 with a brief summary of the nature of the study conducted and the key findings.

**Table 3:** Overview of 3 key papers identified from literature review

**Author**	**Reference**	**Country**	**Research topic**	**Methodology**	**Participant group**	**Analysis**	**Brief overview**	**Key features**
*Kulich, K. R., et al.*	(40)	Sweden	Assessment of patient-centred dentistry in dental phobic patients	Qualitative approach using semi-structured interviews based on video analysis of consultation.	30 interviews conducted with 5 dentists working in a clinic treating anxious patients. Based on 2 separate consultations with 15 patients	Grounded theory	Study to ascertain the key features in delivering PCC to anxious patients attending a specialist clinic in Sweden. Model of care developed based on “holistic perception and understanding of the patient.”	• Communication
								• Empathy
								• Understanding
								• Positive outlook
								• Patient contact
*Loignon, C., et al.*	(41)	Canada	Identify effective approaches to providing care for patients living in poverty	Qualitative approach based on semi-structured interviews	Interviews conducted with 8 dentists with experience of treating patients living in poverty	Thematic analysis	Research to determine what features of PCC are most effective in delivering dental care to people in poverty in Canada. Domains based around socio-humanistic approach.	• Understanding
								• Empathy
								• Non-judgemental
								• Overcome social distance
								• Direct contact
								• Communication
*Scambler, S., et al.*	(42)	UK	Assessment of professional attitudes towards disability in special care dentistry	Qualitative approach based on focus groups & semi-structured interviews	Sample of 30 participants including dentists and staff working within special care dentistry	Retroductive analysis using a theoretical framework	Study to explore attitudes of staff working in Special Care Dentistry towards disability and provision of dental care. Analysis highlighted importance of PCC and revealed key features.	• Holistic approach
								• Individualised care
								• Information/support
								• Time
								• Communication
								• Trust

The three eligible papers all used qualitative research methods and were considered to be of acceptable quality in terms of methodology, analysis, interpretation and relevance when appraised using the CASP framework for qualitative research [38]. A summary sheet providing an overview of the quality assessment is shown in Table 4. None of the studies sampled patients to assess PCC, and instead favoured recruitment of dental care professionals as their participants. All three studies used semi-structured in-depth interviews to collect data, with Scambler [44] also using focus groups. Kulich used video to record consultations, and this information was then used to enrich the data, promote reflection by the dentist involved and stimulate discussion during the interview.

**Table 4:** Quality appraisal of eligible papers identified by literature review

	**Kulich 2003**	**Loignon 2010**	**Scambler 2011**
Was there a clear statement of the aims of the research?	Y	Y	Y
Is a qualitative methodology appropriate?	Y	Y	Y
Was the research design appropriate to address the aims of the research?	Y	Y	Y
Was the recruitment strategy appropriate to the aims of the research?	Y	Y	Limited detail on recruitment/sampling
Were the data collected in a way that addressed the research issue?	Y	Y	Y
Has the relationship between researcher and participants been adequately considered?	Not disclosed	Not disclosed	Not disclosed
Have ethical issues been taken into consideration?	Y	Y	Y
Was the data analysis sufficiently rigorous?	Detail provided on analysis but would appear to have been conducted independently	Y	Lack of detail about who conducted analysis
Is there a clear statement of findings?	Y	Y	Y
How valuable is the research?	Y	Y	Research subject not directly related to literature review but does provide some information on PCC
Reviewers comments	Research highlights 5 facets of delivering a socio-humanistic approach which is consistent with patient-centred care. Recognition that this patient-centred approach had been developed as a consequence of personal experiences and reflection rather than specific training. Study specifically concerned with patients living in poverty, but findings likely to be relevant to delivery of PCC in dentistry in general.	Very interesting paper in terms of research question, methodology and findings. The use of video recording the consultation and then using this as part of the interview process would appear to have enriched the data considerably. Clear theoretical framework described with strong similarities to pre-existing models.	Research focuses on staff attitudes towards delivery of dental care to disabled adults and compares the results against a theoretical framework based on the social model of disability. Key theme to emerge from the interviews is PCC which is described as being “at the heart of the model”. Features of PCC described which focus on a holistic approach, individualised care, communication, trust, information and support.

The first of the three papers, Kulich et al. [42], undertook research to identify the important elements of delivering a patient-centred approach while managing patients with dental anxiety. The research team used qualitative research methods based on semi-structured interviews and video recording of consultations. The participant group included 5 dentists specialising in the treatment of ‘odontophobia’, who were recruited by convenience sampling as part of the dental team working at a clinic treating ‘phobic’ patients in Sweden. Fifteen patients were recruited for treatment from the clinic waiting list and attended on two separate occasions, which provided the background for the interviews with the dentists responsible for their care. This resulted in a total of thirty interviews, which were audio-recorded and transcribed verbatim. The video recording was used to promote discussion and enrich the data provided during the interviews. The video data were not formally analysed, but were used in the style of “action research” to stimulate discussion during the interviews and encourage self reflection and verbalisation on the part of the participant dentist. The authors considered this approach to be beneficial and provided “significant impact” in the generation of data.

Grounded theory was used as the conceptual basis of the study and data were collected until saturation was considered to have been achieved. Content analysis was undertaken and a model of a patient-centred consultation developed. This was defined in terms of one overarching core principle - “*holistic perception and understanding of the patient”* and two underlying categories of *“the dentist’s positive outlook on people”* and *“the dentist’s positive view of patient contact”.* The categories described were underpinned by the following aspects of care: *empathy, equality, dignity, emotional understanding, respect* and *engagement*.

The second paper, by Loignin and co-workers [43], examined the concept of PCC within a different context. They investigated what skills and approaches dentists in Canada used to overcome barriers to dental care for people living in poverty. They undertook a qualitative study with a group of dentists experienced in delivering care to people living in poverty in Canada. Data were collected by conducting semi-structured interviews with 8 dentists identified as a subgroup of an existing research sample. The subgroup was recruited based on their experience of treating patients living in poverty within Montreal. Content analysis was undertaken which revealed three main themes: “*dentists’ experiences with low income patients, perception of poverty and strategies to overcome difficulties with this clientele.”* The authors reported the key aspects of successful delivery of dental care to this group of patients and although they use the term “socio-humanistic approach”, they also refer to this as a “patient-centred approach” within the text. The key features highlighted are:

• *Understanding patients’ social context*

• *Taking time and showing empathy*

• *Avoiding moralistic attitudes*

• *Overcoming social distances*

• *Favouring direct contact with patients*

Finally, Scambler [44] conducted a qualitative study to explore attitudes of staff within Special Care Dentistry towards disability and the provision of dental care. The research was concerned with the social model of disability in terms of dentistry and although the study was not directly concerned with PCC, the findings are closely linked. The authors report that patient-centred care was “at the heart of the model”, and PCC is highlighted as one of the key themes to emerge from the research. The study involved recruitment of thirty staff from a Salaried Dental Service Department in London (England), which included clinicians, dental care professionals and administrative staff. Data were collected through interviews and focus groups and initially analysed thematically. Retroductive analysis was then conducted using a framework based on the social model approach to disability. The authors describe several key features of PCC and detail the importance of a *holistic approach* allowing delivery of *individualised care* by providing *appropriate information and support.* The importance of *trust, time* and *communication* were also highlighted as important factors in providing patient-centred care.

There are a number of similarities between the three papers in terms of the research topic, sampling methods, data collection and findings. However there are contrasting styles in terms of data analysis, particularly between Kulich and Scambler. Kulich used grounded theory to analyse their data and develop theory generation. Data collection, coding and analysis were conducted simultaneously and continued until saturation had been reached. In contrast, Scambler used mid range theory with retroductive analysis. The objective was to undertake theory testing in contrast to Kulich who was theory generating. Although very different in approach, both studies provide insight into the important features of PCC according to the participant samples.

There was a degree of commonality between the findings of the three studies, with each highlighting the need to treat the patient as a person or as an individual in their own right. This reflects the key principles of PCC as described by Gerteis [41]. Each article also reported the importance of a clear focus on delivering a holistic, non-judgemental approach which represented the findings of previous reports on PCC in other areas of healthcare. The importance of communication was highlighted as a key feature of PCC, and this also featured frequently within the rest of the dental literature. The three articles all detailed the importance of breaking down perceived barriers to allow establishment of a dentist-patient relationship through “direct contact”, “patient contact” or “overcoming social distance”.

Two of the eligible papers highlighted the importance of empathy and understanding, which was again representative of the overall dental literature. Other features that were also reported were the importance of information and support, individualised care, trust and the impact of time.

Certain aspects of PCC that feature within the rest of the dental literature have not been described within the evidence-based papers. These include patient satisfaction [45-47], oral health promotion/self care [48] and physical comfort [49].

## Discussion

The results across the three studies show a degree of congruity and tend to highlight similar themes within their findings. This could be indicative of a degree of conformity in terms of PCC across different areas of dentistry, or it could simply be due to the homogenous nature of the participant groups, the study design or the similarities in the area of research investigated.

The studies recruited dentists, dental care professionals and administrative staff to understand the key features of PCC, with none of them engaging with patients directly. This is considered by the authors to be a key finding of this literature review, and highlights the importance of understanding PCC from a “patients” perspective. Health professionals have a wealth of knowledge and experience, but ultimately it is patients’ views, which need to be considered and adequately represented when we wish to understand “patient-centred care”. This has recently been recognised by the Cochrane Collaboration with the importance of involving patients in developing “patient-centred outcome measures” highlighted as a key factor in delivering informed healthcare decisions [50].

The studies were not based in general dental practice and focussed on “specialised’ areas of dentistry concerned with the treatment of vulnerable patients. Although such patients may attend general dental practices for routine care, they are likely to have more specific needs, which are not necessarily representative of the rest of the population. It needs to be recognised that although the features identified within these studies could be highly relevant, there may be distinct differences within a group of patients attending a general dental practice. Kulich et al. suggested in their concluding remarks that their findings may be generalisable within the scope of treating dental phobic patients, but the basic principles “should also be applicable to wider areas of dentistry” when dealing with anxious patients.

The recurring themes within this limited body of literature highlight the importance of a humanistic, non-judgemental approach based around good communication, empathy and understanding. (See Tables 3 & 5) The need to see the patient as a person or as an individual in their own right was a strong theme throughout the research articles and aligns closely to the patient-centred clinical method (PCCM) described by Stewart et al. [31]. This framework is based on the concept that ill health has two components – disease and illness. Stewart et al. recognised that history taking and clinical examination provides information on the disease, but it is equally important to understand and appreciate the patient’s perspective and experience of the illness. An understanding and appreciation of the patient’s feelings can only be achieved by engaging with the patient through communication and developing the patient-doctor (dentist) relationship, and this was highlighted repeatedly within the three articles identified within this review. These concepts also reflect the findings within Kitson’s narrative review of the nursing literature [51] which described three main themes; *patient involvement, relationship* and *context.*

**Table 5:** Comparison of dimensions of patient-centred care

**Dimensions of PCC according to Institute of Medicine**	**Dimensions of PCC within evidence-based dental literature (3 papers)**
1. Compassion, empathy and responsiveness to needs, values and expressed preferences	1. Empathy, equality, dignity, respect, engagement, trust, courtesy, “listened to”, responsive, individualised care, humanistic approach.
2. Co-ordination and integration	2. -
3. Information, communication and education	3. Communication, information.
4. Physical comfort	4. -
5. Emotional support, relieving fear and anxiety	5. Emotional understanding, understood, non-judgemental, positive outlook, patient contact, relationship.
6. Involvement of family and friends	6. (Consent issues)

Despite a close correlation with previous work on PCC, some distinct differences within the dental literature are evident. The most obvious difference was the lack of emphasis within the dental literature on the dimensions of “involvement of family and friends”, “co-ordination and integration” and “physical comfort” as described within the Picker Principles of PCC [5], The NHS Patient Experience Framework [32] and the Institute of Medicine [1].

“Involvement of family and friends” was not considered to be an important feature within dentistry, other than in relation to consent. In contrast, “Involvement of family and friends” is considered one of the most important features of PCC according to Cronin [52] who undertook a review of PCC in 2004.

“Co-ordination and integration” of care was not highlighted within the dental literature as an important feature of PCC, which may reflect the more solitary nature of work as a dentist compared to our medical colleagues who tend to work in larger teams. With the increasing development of skill mix, specialisation and team working, co-ordination and integration is likely to become an increasingly important feature within primary care dentistry.

“Physical comfort” did not feature as an important aspect of patient-centred care within the dental literature. This would appear surprising as fear of pain is considered to be a common barrier to dental attendance for a significant proportion of the population. As highlighted previously, sampling for the three research articles was restricted to dental care professionals with no patients selected as participants. It is conceivable that this aspect of patient-centred care may have featured if patients had been included within the interviews.

These differences highlight the importance of understanding the features of PCC within the contexts of setting, sampling and the area of health investigated. The dimensions described by the Picker Institute [5], the Institute of Medicine [1] and The Kings Fund [3] focus on the secondary care setting, predominately with in-patient care. This would explain the importance of “involvement of family and friends” and “co-ordination and integration”, which would be less relevant to the delivery of dental care in a general dental practice.

Little et al [24] investigated patient preferences in attending general medical practice, based on a questionnaire designed to assess patient-centredness. The authors identified three important domains of patient-centredness from the patients’ perspective: *communication, partnership*, and *health promotion*. Communication and partnership were key aspects reported in the dental literature, although health promotion did not feature. General dental practice may be considered to be more closely aligned to general medical practice than it is to secondary care in terms of continuity of care, familiarity and their role within the community. This will obviously be dependent on the practice, the setting and the healthcare system. There are, however, many differences between general dental practice and medical practice and one should not assume that the key features of PCC would be the same for both.

The importance of communication and relationship was highlighted repeatedly throughout the dental literature and would appear to be a cornerstone of PCC. The overarching theme of the features reported was around the importance of “soft skills” of the clinician. Empathy, equality and emotional understanding were considered to be particularly important and this would appear to support the work of Burke and Croucher [53], and Holt and McHugh [54] on patients’ views of the important features of a good dentist.

The primary aim of the general dental practitioner is to improve and maintain the oral health of their patients and this is normally based on a long term relationship rather than episodic care [55]. This relationship is crucial and success is based on trust, respect and mutual understanding [56,57]. Communication is key, but must not be considered as simply an act of giving and receiving information. Communication is about establishing a “connection” on a human level in order that we can understand the beliefs, needs and preferences of the individual. This is an important theme, which has been repeatedly identified within this literature review and highlights the importance of “putting the patient first” and taking a holistic approach to care.

## Conclusions

Delivery of patient-centred care is an important aspect of providing quality dentistry, but there appears to be a poor understanding of the term within the existing literature. This paper demonstrates that there is only limited evidence to provide an understanding of patient-centred care within dentistry and the research, which has been published, does not relate to general dental practice.

The NHS Patient Experience Framework states that “*it is possible to apply a single generic framework....to a wide range of health conditions and settings*.” [32] The results of this review, albeit from a limited evidence-base, would appear to indicate that there are some distinct differences within the domains of PCC in dentistry when compared to other areas of health. In view of this it may not be appropriate to simply use a generic medical framework to assess PCC as an effective indicator of quality in dentistry.

These findings are equally relevant on an international perspective, for countries striving to implement quality management systems within general dental practice. This lack of evidence-based research must be addressed if we wish to measure PCC within general dental practice and deliver quality improvement. Research needs to be undertaken within primary care and must be developed from a patients’ perspective if we wish to understand patient-centred care within dentistry.

### Key findings

• Future research should ensure that a patient’s perspective of PCC is adequately represented.

• There is a lack of evidence to adequately understand PCC within dentistry.

• The evidence which is currently available on PCC in dentistry is not necessarily generalisable to general dental practice.

• Further research is necessary to understand the key features of PCC within general dental practice if we wish to use this as a quality indicator.

## Competing interest

The authors declare that they have no competing interests.

## Authors’ contributions

IJM was involved in all aspects of this review including the literature search, data extraction, quality appraisal and drafting the manuscript. CC provided advice, support and direction on the search strategy and contributed to the final draft of the paper. DRM, JF and EJK provided advice and support on all aspects of the systematic review including development of the protocol, screening, data extraction and editing the paper. DRM and JF were directly involved with quality appraisal, synthesis of the data and interpretation of the results. All authors read and approved the final manuscript.

## Pre-publication history

The pre-publication history for this paper can be accessed here:

http://www.biomedcentral.com/1472-6831/14/64/prepub

## Supplementary Material

Additional file 1 Search strategy. Click here for file

Additional file 2 Search terms used within literature review. Click here for file

## References

[B1] Institute of Medicine: Crossing the Quality Chasm Crossing the Quality Chasm: A new health system for the 21st century Book Crossing the Quality Chasm: A new health system for the 21st century 2001 City: Institute of Medicine 11549951

[B2] Australian Commission on Safety and Qaulity in Healthcare Patient-Centred Care: Improving Quality and Safety by Focusing Care on Patients and Consumers Book Patient-Centred Care: Improving Quality and Safety by Focusing Care on Patients and Consumers 2010 City: ACSQHC

[B3] Goodrich J Cornwell J Seeing the Person in the Patient. The Point of Care Review Paper 2008 London: King’s Fund

[B4] Agency for Healthcare Research and Quality (AHRQ) National Healthcare Quality Report Book National Healthcare Quality Report 2003 City: AHRQ

[B5] Eight picker principles of patient-centered care http://pickerinstitute.org/about/picker-principles/

[B6] Saha S Beach MC Cooper LA Patient centeredness, cultural competence and healthcare quality J Natl Med Assoc 2008 100 1275 1285 19024223 10.1016/s0027-9684(15)31505-4 PMC2824588

[B7] Epstein RM Mauksch L Carroll J Jaén CR Have you really addressed your patient’s concerns? Fam Pract Manag 2008 15 35 40 18422265

[B8] Stewart M Brown JB Donner A McWhinney IR Oates J Weston WW Jordan J The impact of patient-centered care on outcomes J Fam Pract 2000 49 796 804 11032203

[B9] Bertakis KD Azari R Patient-centered care is associated with decreased health care utilization J Am Board Fam Med 2011 24 229 239 10.3122/jabfm.2011.03.100170 21551394

[B10] Bertakis K KD Rahman A Determinants and outcomes of patient-centered care Patient Educ Couns 2011 85.1 46 52 20801601 10.1016/j.pec.2010.08.001

[B11] Irwin RS Richardson ND Patient-focused care: using the right tools Chest 2006 130 73S 82S 10.1378/chest.130.1_suppl.73S 16840370

[B12] Professor the Lord Darzi of Denham K High Quality Care For All. NHS Next Stage Review Final Report Book High Quality Care For All. NHS Next Stage Review Final Report 2008 London: The Stationary Office

[B13] Information Centre High Quality Care for All. Measuring for Quality Improvement Book High Quality Care for All. Measuring for Quality Improvement 2009 London: The Stationary Office

[B14] Greenhalgh J The applications of PROs in clinical practice: what are they, do they work, and why? Qual Life Res 2009 18 115 123 10.1007/s11136-008-9430-6 19105048

[B15] Palfreyman S Patient-reported outcome measures and how they are used Nurs Older People 2011 23 31 36 21323049 10.7748/nop2011.02.23.1.31.c8295

[B16] European Regional Organisation of the Federation Dentaire Internationale WG Quality in Dentistry Report 2007–2010 Book WG Quality in Dentistry Report 2007–2010 2010 City: FDI World Dental Federation

[B17] Dental Quality Alliance Quality Measurement in Dentistry: A Guidebook Book Quality Measurement in Dentistry: A Guidebook 2012 City: American Dental Association

[B18] Milsom KM Threlfall A Pine K Tickle M Blinkhorn AS Kearney-Mitchell P The introduction of the new dental contract in England – a baseline qualitative assessment Br Dent J 2008 204 59 62 10.1038/bdj.2008.1 18223578

[B19] Chestnutt IG Davies L Thomas DR Practitioners’ perspectives and experiences of the new National Health Service dental contract Br Dent J 2009 206 E18 10.1038/sj.bdj.2009.354 19390531

[B20] Committee THoCHS Report on Dental Services. Fifth Report of Session 2007–2008 Book Report on dental services. Fifth Report of Session 2007–2008 2008 City: The Stationary Office

[B21] Davies BJB Macfarlane F Clinical decision making by dentists working in the NHS General Dental Services since April 2006 Br Dent J 2010 209 E17 E17 10.1038/sj.bdj.2010.1080 21109782

[B22] NHS ENGLAND Dental Care and Oral Health - call to action [Online] 2014 London: NHS England Available: http://www.england.nhs.uk/ourwork/qual-clin-lead/calltoaction/dental-call-to-action/ [Accessed 1st May 2014]

[B23] Department of Health Dental Quality and Outcomes Framework Book Dental Quality and Outcomes Framework 2011 City: The Stationary Office

[B24] Little P Everitt H Williamson I Warner G Moore M Gould C Ferrier K Payne S Preferences of patients for patient centred approach to consultation in primary care: observational study BMJ 2001 322 468 10.1136/bmj.322.7284.468 11222423 PMC26564

[B25] Mead N Bower P Patient-centredness: a conceptual framework and review of the empirical literature Soc Sci Med 2000 51 1087 1110 10.1016/S0277-9536(00)00098-8 11005395

[B26] Robinson JH Callister LC Berry JA Dearing KA Patient-centered care and adherence: definitions and applications to improve outcomes J Am Acad Nurse Pract 2008 20 600 607 10.1111/j.1745-7599.2008.00360.x 19120591

[B27] Stewart M Towards a global definition of patient centred care BMJ 2001 322 444 445 10.1136/bmj.322.7284.444 11222407 PMC1119673

[B28] Planetree Foundation The planetree model*,* [Online] 2011 Available: http://planetree.org/about-planetree/ [Accessed 27th May 2014]

[B29] Little P Everitt H Williamson I Warner G Moore M Gould C Ferrier K Payne S Observational study of effect of patient centredness and positive approach on outcomes of general practice consultations BMJ 2001 323 908 911 10.1136/bmj.323.7318.908 11668137 PMC58543

[B30] Mead N Bower P Patient-centred consultations and outcomes in primary care: a review of the literature Patient Educ Couns 2002 48 51 61 10.1016/S0738-3991(02)00099-X 12220750

[B31] Stewart M Brown JB Weston WW McWhinney IR McWilliam C Freeman T Patient-Centered Medicine: Transforming the Clinical Method 1995 London: Sage

[B32] NHS patient experience framework http://www.dh.gov.uk/health/2012/02/patient-experience-framework/

[B33] Khan KS Ter Riet G Glanville J Sowden A Kleijnen J Undertaking Systematic Reviews of Research on Effectiveness: CRD’s Guidance for Carrying out or Commissioning Reviews. No. 4 2001 2 York: NHS Centre for Reviews and Dissemination

[B34] Papaioannou D Sutton A Carroll C Booth A Wong R Literature searching for social science systematic reviews: consideration of a range of search techniques Health Info Libr J 2010 27 114 122 20565552 10.1111/j.1471-1842.2009.00863.x

[B35] Greenhalgh T Peacock R Effectiveness and efficiency of search methods in systematic reviews of complex evidence: audit of primary sources BMJ 2005 331 1064 1065 10.1136/bmj.38636.593461.68 16230312 PMC1283190

[B36] Balint E The possibilities of patient-centered medicine J R Coll Gen Pract 1969 17 269 276 5770926 PMC2236836

[B37] Irving MJ Tong A Jan S Cass A Rose J Chadban S Allen RD Craig JC Wong G Howard K Factors that influence the decision to be an organ donor: a systematic review of the qualitative literature Nephrol Dial Transplant 2012 27 2526 2533 10.1093/ndt/gfr683 22193049

[B38] CASP An appraisal tool for qualitative research http://wwwphrunhsuk/casp/qualitativepdf (Last accessed 2 September 2013)

[B39] Masood M Thaliath ET Bower EJ Newton JT An appraisal of the quality of published qualitative dental research Community Dent Oral Epidemiol 2011 39 193 203 10.1111/j.1600-0528.2010.00584.x 21070318

[B40] Tong A Sainsbury P Craig J Consolidated criteria for reporting qualitative research (COREQ): a 32-item checklist for interviews and focus groups Int J Qual Health Care 2007 19 349 357 10.1093/intqhc/mzm042 17872937

[B41] Gerteis M Edgeman-Levitan S Daley J Delbanco TL Through the Patient’s Eyes 1993 San Francisco: John Wiley & Sons

[B42] Kulich KR Berggren U Hallberg LR A qualitative analysis of patient-centered dentistry in consultations with dental phobic patients J Health Commun 2003 8 171 187 10.1080/10810730305694 12746040

[B43] Loignon C Allison P Landry A Richard L Brodeur JM Bedos C Providing humanistic care: dentists’ experiences in deprived areas J Dent Res 2010 89 991 995 10.1177/0022034510370822 20525962

[B44] Scambler SL Emma Z Liana G Jennifer E Professional attitudes towards disability in special care dentistry J Disabil Oral Health 2011 12 51 58

[B45] Roskell CW Deborah; Bonner, Cathy; Fairchild, Roseanne [Commentary on] Developing patient-centred care in health professionals: reflections on introducing service-learning into the curriculum Int J Therapy Rehabil 2012 19 448 457 10.12968/ijtr.2012.19.8.448

[B46] McNair A Gardiner P Sandy JR Williams AC A qualitative study to develop a tool to examine patients’ perceptions of NHS orthodontic treatment J Orthod 2006 33 97 106 10.1179/146531205225021483 16751431

[B47] Chapple H Shah S Caress AL Kay EJ Exploring dental patients’ preferred roles in treatment decision-making - a novel approach Br Dent J 2003 194 321 327 10.1038/sj.bdj.4809946 12682659

[B48] Bauer J Spackman S Chiappelli F Prolo P Interdisciplinary resources optimize evidence-based dental practice J Evid Based Dent Pract 2005 5 67 73 10.1016/j.jebdp.2005.04.003 17138335

[B49] Brennan M Houston F O’Sullivan M O’Connell B Patient satisfaction and oral health-related quality of life outcomes of implant overdentures and fixed complete dentures Int J Oral Maxillofac Implants 2010 25 791 800 20657876

[B50] Gartlehner G Maria F Is the cochrane collaboration prepared for the era of patient-centred outcomes research? Cochrane Database Syst Rev 2013 3 ED000054 23641478 10.1002/14651858.ED000054 PMC10846354

[B51] Kitson A Marshall A Bassett K Zeitz K What are the core elements of patient-centred care? A narrative review and synthesis of the literature from health policy, medicine and nursing J Adv Nurs 2013 69 4 15 10.1111/j.1365-2648.2012.06064.x 22709336

[B52] Cronin C Patient-Centered Care: An Overview of Definitions and Concepts Book Patient-Centered Care: An Overview of Definitions and Concepts 2004 City: National Health Council

[B53] Burke L Croucher R Criteria of good dental practice generated by general dental practitioners and patients Int Dent J 1996 46 3 9 8744911

[B54] Holt VP McHugh K Factors influencing patient loyalty to dentist and dental practice Br Dent J 1997 183 365 370 10.1038/sj.bdj.4809512 9419943

[B55] Li RW Chow TW The specialty of family dentistry: a future for general dental practitioners? Dent Update 2004 31 6 7 15000002 10.12968/denu.2004.31.1.6

[B56] Newsome PR McGrath C Patient-centred measures in dental practice: 3. Patient satisfaction Dent Update 2007 34 87 90 17432772 10.12968/denu.2007.34.2.87

[B57] Sbaraini A Carter SM Evans RW Blinkhorn A Experiences of dental care: what do patients value? BMC Health Serv Res 2012 12 177 177 10.1186/1472-6963-12-177 22726888 PMC3407476

