# Supplementary figures and images for: Patient-centred care in general dental practice - a systematic review of the literature
Source: BMC Oral Health. 2014 Jun 5;14:64. doi: 10.1186/1472-6831-14-64 (PMC4054911; doi:10.1186/1472-6831-14-64)

## Appendix 1

### Search strategy

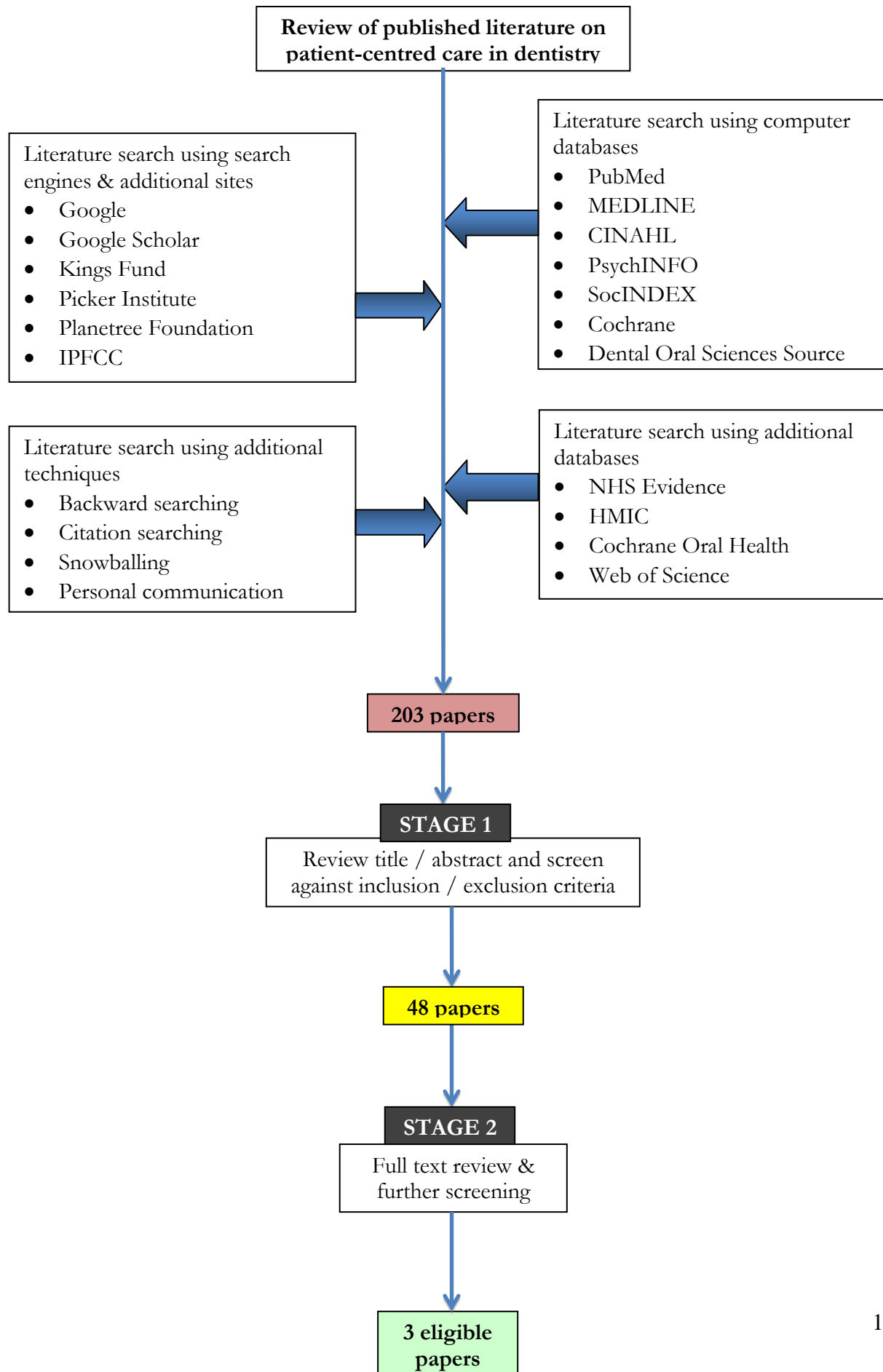

Supplement: Additional file 1 — Search strategy. [file 1472-6831-14-64-S1.pdf]
