# Supplementary material for: Patient-centred care in general dental practice - a systematic review of the literature
Source: BMC Oral Health. 2014 Jun 5;14:64. doi: 10.1186/1472-6831-14-64 (PMC4054911; doi:10.1186/1472-6831-14-64)
Supplement: Additional file 2 — Search terms used within literature review. [file 1472-6831-14-64-S2.pdf]

Appendix 2

Search terms used within literature review

**Search Terms**	**Search**
1. ("patient centred care"[Tiab] OR "patient centered care"[Tiab] OR "person centered care"[Tiab] OR "person centred care"[Tiab] OR "patient focused care"[Tiab] OR "person focused care"[Tiab])	**(2,000)**
2. (dental[Tiab] OR oral[Tiab] OR dentist*[Tiab])	**(440,216)**
3. "Patient-centred care”[Mesh]	**(8,572)**
4. "Dentistry"[Mesh]	**(207,683)**
5. 1 **OR** 3	**(9,539)**
6. 2 **OR** 4	**(563,809)**
7. 5 **AND** 6	**(162)**
